# Preparation and Orthogonal Analysis for Dual-Responsive Electrochromic Polymer Dispersed Liquid Crystal Devices

**DOI:** 10.3390/polym15081860

**Published:** 2023-04-13

**Authors:** Haiyu Xian, Lin Li, Yilei Ding, Mingjing Chu, Changqing Ye

**Affiliations:** School of Materials Science and Engineering, Suzhou University of Science and Technology, Suzhou 215009, China

**Keywords:** polymer dispersed liquid crystals, electrochromic, dual-responsive, orthogonal analysis

## Abstract

In this work, we provide a fabrication method for dual-responsive electrochromic (EC) polymer dispersed liquid crystal (PDLC) devices. The EC PDLC device was developed by combing the PDLC technique and a colored complex formed via a redox reaction without a specific EC molecule in a simple preparation method. The mesogen played dual roles in the device for scattering in the form of microdroplets and participating in the redox reactions. Orthogonal experiments were performed with the acrylate monomer concentration, the ionic salt concentration, and the cell thickness as factors to investigate the electro-optical performance for the achievement of optimized fabrication conditions. The optimized device presented four switchable states modulated by external electric fields. The light transmittance of the device was changed by an alternative current (AC) electric field while the color change was realized by a direct current (DC) electric field. Variations of mesogen and ionic salt species can modulate the color and hue of devices, which solves the disadvantage of a single color for traditional EC devices. This work lays the foundation for realizing patterned multi-colored patterned displays and anti-counterfeiting via screen printing and inkjet printing techniques.

## 1. Introduction

A liquid crystal (LC) is an intermediate state between the solid state and the liquid state, which demonstrates the anisotropy and fluidity similar to that of crystals and liquids, respectively [1]. LCs are able to respond to various external stimuli (heat, electricity, light, and magnet force) resulting in the rearrangement of the anisotropic LC molecules (mesogens) accompanied by variations in optical properties, which demonstrates substantial significance for diverse applications, such as displays [2,3,4], optics [5], photonics [6], biosensors [7,8], and biomedicines [9]. For example, cholesteric liquid crystals (CLCs) have been recently reported to switch within sub-microsecond by electrically modifying the orientational order of molecules and quenching their fluctuations [10]. In addition, a novel CLC-based device has been proposed for electrically active and thermally passive smart windows [11]. Its electrically active mode was due to the reversible transmission modulated by applied AC voltage while the passive mode was based on the controllable strength of voltage-induced electrohydrodynamic flow and temperature-dependent dynamic scattering for passive control. Additionally, LC devices with low-voltage driven scattering-controllability [12] and double-layer structures of dual-frequency [13] have been explored for smart windows as well. The introduction of polymers into liquid crystals endows the liquid crystal composite systems with expanded functionalities and applications, such as among which polymer dispersed liquid crystals (PDLCs) have attracted considerable attention on account of the excellent electro-optical properties and applications [14,15,16]. In a PDLC, LC microdroplets are dispersed in the polymer matrix, which is generated by the phase separation of a homogeneous mixture containing monomers and LCs via the polymerization with the treatment of heat or UV irradiation [17]. Sandwiching a PDLC between two electrodes will generate a PDLC device capable of switching light. Basically, the mesogenic optical axis in the microdroplet is randomly oriented resulting in a strong light scattering without any electric field (off state) while mesogens are forced to rearrange depending on the direction and strength of the electric field to induce an optical change in the transmittance with an AC electric field applied (on state). Electro-modulation of the refractive index between the mesogens and the polymer matrix realizes the transmittance variation of PDLCs [18,19].

At present, PDLCs demonstrate tremendous potential for a variety of applications including physical and chemical sensors [20,21,22], displays [23,24], time temperature indicators [25], smart windows [26], etc. The electro-optical performance of PDLCs has been improved in various strategies, such as doping guests (e.g., dyes [27] and nanoparticles [14,28]), utilizing LC elastomers [29], and innovating novel modes [30,31,32]. However, the singleness of the function and color of common PDLCs largely limits their application scope. In essential, PDLCs with different colors can be obtained by doping dyes [33], but the presence of dyes can absorb ultraviolet light and affect the photopolymerization process with monomers unreacted resulting in uncontrollable morphologies and properties of the PDLC device [34]. Electrochromic (EC) devices are able to switch color in response to the external electrical field with a general configuration of EC materials and electrolytes sandwiched between conductive electrodes [35]. Reversible color modulation is achieved through a stable and redox reaction with a low external driving voltage [36,37]. EC materials can be directly doped into PDLCs to broaden the discoloration characteristics of corresponding devices [38]. However, the composition is complex, and the process is tedious due to the addition of extra discoloration materials and electrolytes. Nakamura [39,40] observed the color change in both the LC phase and the isotropic state under a DC electric field when doping a small amount of an electrolyte, e.g., a tetraalkylammonium iodide into LCs, which was due to colored complexes produced between the electrolyte and mesogens once cations migrated to the cathode. Additionally, modifying the end group of mesogens can alter the color of the formed complex [41]. Therefore, combining the PDLC feature with the redox reaction between mesogens, and ionic salts turns out to be an effective approach to solving the single-function problem that bothers PDLCs, which can avoid incomplete polymerization and inhomogeneous coloration induced by doped dyes and prevent the leakage of components and the damage of devices due to the confinement of polymer network.

**Scheme 1:**
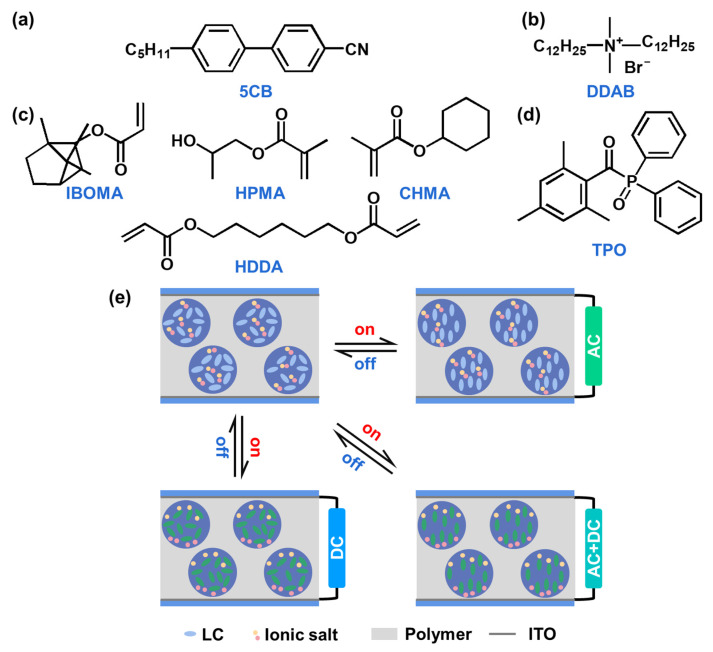
(**a**–**d**) Related chemical structures and (**e**) schematic illustration for the working principle of the EC PDLC.

In this work, we present a dual-responsive EC PDLC device featured with polychromic modulation fabricated by photopolymerization-induced phase separation. The device demonstrates four states, which can be switched by tuning the light transmittance by the AC electric field and the coloration by the DC electric field, respectively (Scheme 1). The bifunctional mesogens act not only as mesogens to modulate light transmittance but also as chromogens for coloration. The coloration is simply achieved by the redox reaction with the coloration from colorless to green between a common nematic mesogen, 4-cyano-4′-pentylbiphenyl (5CB), and an electrolyte, didodecyldimethylammonium bromide (DDAB), under a DC electric field without additional EC materials. The influence of the DDAB concentration on the coloration performance of the device was investigated. The optimized formulation for the device was explored with an orthogonal experiment by comprehensively taking the electro-optical characteristics and EC properties of the device into consideration. The threshold and saturation voltages of the device were much lower than the safe voltage of the human body. Additionally, a diversity of electrochemistry-induced coloration behaviors could be achieved by varying the type of mesogens and electrolytes. This work lays the foundation for the realization of the patterned display through printing methods, such as screen printing and inkjet printing technology, and provides a novel idea for anti-counterfeiting applications.

## 2. Materials and Methods

### 2.1. Materials

Liquid crystal 4-Cyano-4′-pentylbiphenyl (5CB, 99.8%) was purchased from Yantai Xianhua Technology Group Co., Ltd (Yantai, China). Acrylate monomers including isobornyl methacrylate (IBOMA), hydroxypropyl methacrylate (HPMA), cyclohexyl methacrylate (CHMA), 1,6-hexanediol diacrylate (HDDA), diphenyl(2,4,6-trimethylbenzoyl)phosphine oxide (TPO), and didodecyldimethylammonium bromide (DDAB) were purchased from Aladdin (Shanghai, China). Norland optically curable adhesive NOA61 was purchased from Thorlabs, Inc. (Newton, NJ, USA) Chemicals were used as received unless otherwise noted. All glassware used for the reactions was dried overnight in an oven at 140 °C. Deionized water was used in the experiments throughout.

### 2.2. Preparation Method

The electrochromic PDLC devices were prepared by photopolymerization-induced phase separation under ultraviolet radiation. An acrylate monomer mixture composed of IBOMA (35 wt%), HPMA (30 wt%), CHMA (30 wt%), and HDDA (5 wt%), was applied throughout this work with TPO (0.6 wt%) as the photoinitiator. The acrylate monomer mixture, TPO, and 5CB were added into a clean container and magnetically stirred at room temperature followed by addition of DDAB to result in a homogeneous mixture. Then, the mixture was transferred to a glass cell through a capillary force and cured by continuous ultraviolet irradiation (wavelength: 365 nm, power density: 3 mW/cm^2^) for 30 min at room temperature. DDAB-doped LC samples were prepared in similar method but without the addition of the acrylate monomer mixture and TPO. Empty glass cells were prepared by gluing two pieces of indium tin oxide (ITO) coated glass with the thickness controlled by spacers with different diameters (5 μm, 10 μm, 15 μm, and 20 μm).

### 2.3. Measurements

A white light source was applied to the sample and the transmitted optical signal was collected by a fiber optic spectrometer NOVA (Shanghai Fuxiang Optics Co., Ltd., Shanghai, China). Light transmittance of an empty cell was normalized as 100%. Test parameters including off-state transmittance (T_off_), on-state transmittance (T_on_), contrast ratio (CR), threshold voltage (V_th_), saturation voltage (V_sat_), and absorption were recorded to evaluate the device performance. The sample morphology was observed by a Nikon microscope LV100NPOL.

## 3. Results

**Figure 1:**
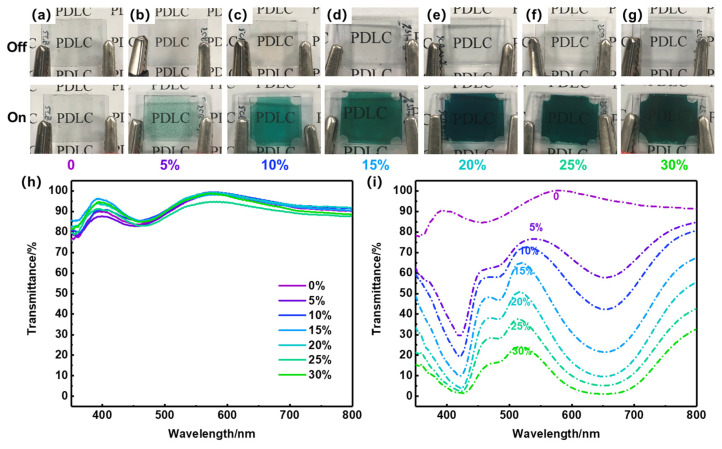
Photographic images of cell samples composed of 5CB and DDAB with different DDAB concentrations without (top row) and with (bottom row) a DC voltage of 4.5 V applied: (**a**) 0, (**b**) 5 wt%, (**c**) 10 wt%, (**d**) 15 wt%, (**e**) 20 wt%, (**f**) 25 wt%, and (**g**) 30 wt% and corresponding light transmittance spectra for (**h**) off and (**i**) on states.

The electrochromic property of DDAB-doped LC samples was initially investigated. Homogeneous mixtures containing 5CB and DDAB with various DDAB concentrations (0 wt%, 5 wt%, 10 wt%, 15 wt%, 20 wt%, 25 wt%, and 30 wt%) were prepared and then sandwiched between two pieces of ITO glass with a thickness of 20 μm. The resultant cell samples were transparent and colorless (Figure 1a–g, top row) and displayed similar transmittance (Figure 1h) without any electrical field (off state). With a DC voltage of 4.5 V applied (on state), all cells displayed green color in appearance with different transparencies (Figure 1a–g, bottom row) and their transmittance spectra demonstrated two absorption bands at 422 nm and 658 nm (Figure 1i). The absorption at 422 nm was more intense, which contributed to the physical green color of cell samples under the DC electric field. In the following study, light transmittance at 422 nm was utilized to evaluate the performance of samples. As shown in the bottom row from Figure 1a–g, the sample appearance color varied from light green to dark green as the concentration of DDAB raised, which was in accordance with the transmittance spectra that the transmittance decreased significantly from the initial T_on_ value of 87% (pure 5CB) to final T_on_ of 2% (30 wt% DDAB). With the DC electric field removed (off state), all samples returned to the transparent colorless state, which exhibited responsive abilities to reversibly switch color by a DC electric field. In the on state, ammonium cations (NH_4_^+^) and bromide anions (Br^−^) in the sample migrated to the cathode and anode, respectively [41]. At the cathode, NH_4_^+^ reacted with the benzene ring from 5CB to form a green free radical oxide while at the anode bromine was generated. A higher NH_4_^+^ concentration, namely DDAB, would accumulate more free radical oxides and a stronger transmittance in the green region. In the off state, the cell was bleached due to the recombination of the halide molecule and with the green free radical oxide. Since the sample with 5 wt% DDAB was inhomogeneous and the sample with 30 wt% DDAB was too sticky, the weight ration of DDAB to 5CB for the following study was controlled from 10 wt% to 25 wt%.

EC PDLC devices based on 5CB, the acrylate monomer mixture, and DDAB were further investigated to explore the constitution with the best performance. The introduction of acrylate monomers can reduce the viscosity of samples. Additionally, the increased functionality of acrylates is beneficial to the formation of a cross-linked network with an improved cross-linking degree and an enhanced polymer strength, which, however, can raise the anchoring force of mesogens resulting in a larger driving voltage. Therefore, it is necessary to define an appropriate dosage of acrylate monomers. The thickness of LC cells also affects the electro-optical performance of PDLCs. In a thin LC cell, only a small number of meshes exist in the polymer network, which usually easily leads to the generation of large mesh size and an uneven distribution with respect to the polymer network. Accordingly, the resultant LC microdroplets exhibit a small number but large size and poor homogeneity. In the off state, such a PDLC device is generally suffering from light leakage due to the attenuated scattered light intensity by LC microdroplets, which can present a larger T_off_ value. Additionally, LC cells with too thick thickness are not welcomed since those PDLCs require large threshold voltage values to be working. As a result, an appropriate cell thickness is necessary for a high-performance and low-energy consumed PDLC. Moreover, the EC property is intimately linked to the concentration of DDAB. To achieve the optimal parameters for an EC PDLC device with a reduced quantity of work, an orthogonal experiment method was introduced in this study. We designed a factor-level table with three factors, A (the weight ratio of the acrylate monomer mixture to 5CB), B (the weight ratio of the ammonium salt to 5CB), and C (cell thickness), and four levels (Ⅰ, Ⅱ, Ⅲ, and Ⅳ) as shown in Table 1. To be specific, the weight ratio of the acrylate monomer mixture to 5CB (A) was from 33.4 wt% to 66.7 wt% while the weight ratio of the ammonium salt to 5CB (B) was from 11.2 wt% to 33.4 wt%. The cell thickness was controlled as 5 μm, 10 μm, 15 μm, and 20 μm, respectively. The following experiments were performed experiments based on the appropriate orthogonal table generated by Software Minitab 21 (Taguchi Design, Japan), which displayed the fabrication constitutions for sixteen EC PDLC samples with three variables controlled as shown in Table 2. Based on the experimental results, range analysis was applied to determine the factors’ sensitivity.

**Figure 2:**
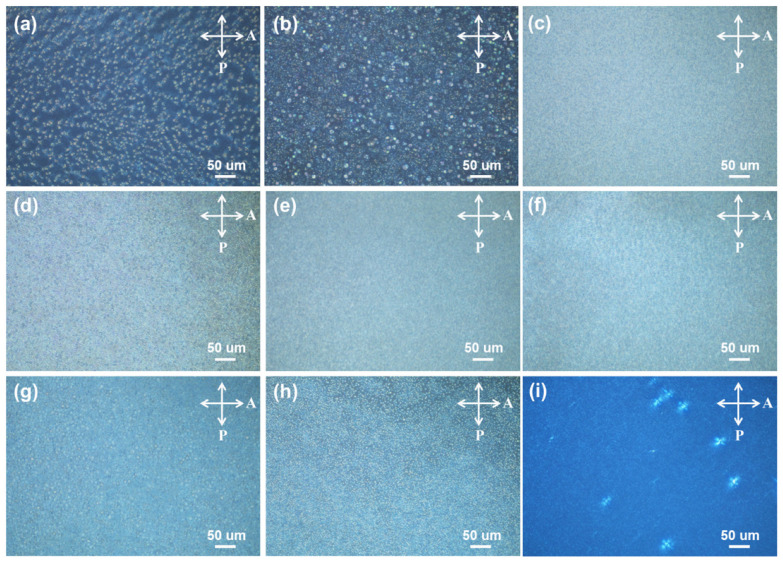
POM images of (**a**) 1, (**b**) 2, (**c**) 5, (**d**) 6, (**e**) 7, (**f**) 9, (**g**) 10, (**h**) 11, and (**i**) 12 observed under crossed polarizers with a scale bar of 50 μm.

Samples were prepared according to the fabrication constitution shown in Table 2. Once fabricated, **1**, **2**, **5**, **6**, **7**, **8**, **9**, **10**, and **11** displayed an opaque color while samples **3**, **4**, **12**, **13**, **14**, **15**, and **16** were transparent colorless, which meant the absence of the PDLC feature. All samples were observed with a polarized optical microscope (POM) under crossed polarizers. The POM images of **1**, **2**, **5**, **6**, **7**, **8**, **9**, **10**, and **11** (Figure 2a–h) clearly presented the existence of LC microdroplets but with various diameters. Samples **1** and **2** exhibited similar POM images (Figure 2a,b), which displayed LC microdroplets with larger diameters than those of other samples. In addition, the LC microdroplets in **1** and **2** were of poor monodispersity and small density in the view. POM images of **5**, **6**, **7**, **8**, and **9** showed densely distributed LC microdroplets with relatively small diameters (Figure 2c–f). For samples without the PDLC feature, no LC microdroplet was found under POM. For instance, the POM image of **12** (Figure 2i) presented no existence of LC microdroplets but few typical Schlieren textures in the view. For **13**–**16**, the disappearance of LC microdroplets was due to the high concentration of monomers and the low concentration of 5CB, which would result in the disability of light modulation. The absence of PDLC features for **4** and **12** was probably ascribed to the high concentration of the ammonium salt. The synergetic effect of monomer and ammonium salt concentration might decide the morphological and optical properties of **3**. Therefore, **1**, **2**, **5**, **6**, **7**, **8**, **9**, **10**, and **11** would be discussed in the following study.

**Figure 3:**
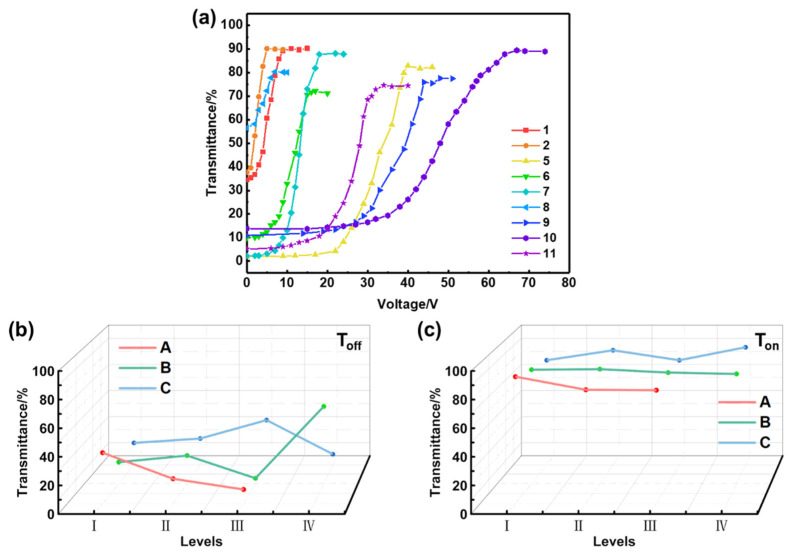
(**a**) The voltage-dependent transmittance of **1**, **2**, **5**, **6**, **7**, **8**, **9**, **10**, and **11**. Range analysis charts of (**b**) T_off_ and (**c**) T_on_ with different levels of three factors A, B, and C.

The electro-dependent transmittance of **1**, **2**, **5**, **6**, **7**, **8**, **9**, **10**, and **11** was further characterized under an AC electric field (Figure 3). All those samples were opaque in appearance in the beginning, which corresponded to the low transmittance values at the AC voltage of 0, namely the off state transmittance (T_off_), in Figure 3. **1**, **2**, and **8** clearly showed higher T_off_ values than others, which was consistent with their POM images that showed larger LC microdroplets. As the AC voltage raised, their transmittance gradually increased differently. In essence, the randomly distributed mesogens in the microdroplets among the polymer network cause intensive light scattering leading to an opaque color without any electrical field. However, applying an AC electric field will orientationally rearrange mesogens according to the direction of the electric field resulting in a transparent appearance. At the same AC voltage value, those samples displayed different transmittance values due to the variation in the cell constitution. The orthogonal range analysis method was employed to add values at the same level under the same factor to generate the average value at each level, on which the range analysis diagram was based (Figure 3b,c). According to the difference between the largest and smallest T_off_ values, the influence sequence regarding T_off_ was B > A > C (Figure 3b). The presence of ammonium salt affected the phase separation process. Based on the experimental results, once the DDAB concentration of the sample was above 33.4%, no electro-dependent optical switching could be observed. The monomer concentration affected the formation of the intermingled polymer network. As the monomer concentration raised, the polymer network became more compact, which added difficulty in the diffusion of mesogens and the formation of large LC microdroplets and accordingly attenuated T_off_ values. Values of T_on_ for those samples were basically in the range between 80–90% with inconspicuous changes (Figure 3c). Thus, it was difficult to decide the impacting power for the three factors A, B, and C.

**Figure 4:**
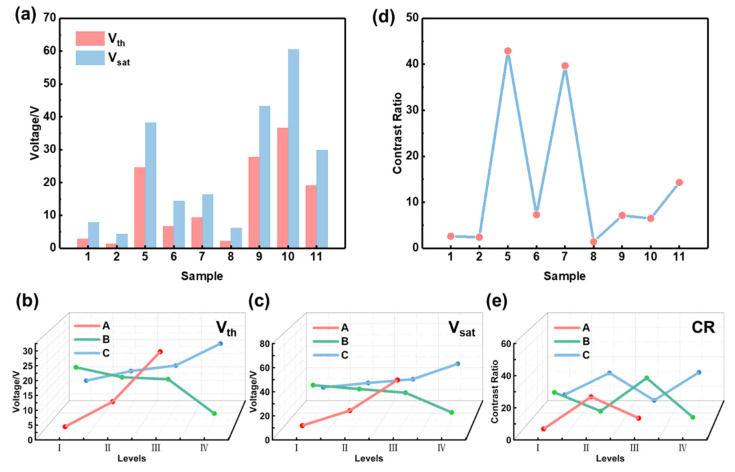
(**a**) Histogram of the threshold voltage (V_th_) and the saturation voltage (V_sat_) and (**d**) contrast ratio of **1**, **2**, **5**, **6**, **7**, **8**, **9**, **10**, and **11**. The corresponding range analysis charts of (**b**) V_th_, (**c**) V_sat_, and (**e**) CR values with different levels of three factors A, B, and C.

Based on the transmittance spectra, the threshold voltage (V_th_) and saturation voltage (V_sat_) were further analyzed (Figure 4a). Sample **2** had the smallest V_th_ and V_sat_ values of 1.3 V and 4.3 V, respectively. Sample **10** had the largest V_th_ and V_sat_ values of 36.6 V and 60.5 V, respectively, which exceeded the safe voltage of the human body (36 V). According to the orthogonal range analysis, values at the same level under the same factors were added up to calculate the average values at each level resulting in the range analysis diagram (Figure 4b,c). In general, the monomer concentration (A) and the cell thickness (C) had a positive correlation with both V_th_ and V_sat_ values of samples. It was clearly exhibited that the monomer concentration had the greatest effect on V_th_ and V_sat_ values. As the monomer concentration increased, the diameter of the LC microdroplet became smaller, in which case the interface of the polymer network displayed a stronger anchoring force towards LC microdroplets and accordingly higher energy (voltage), e.g., V_th_ and V_sat_, was required to drive the mesogenic rearrangement. With the same voltage applied, the electric field strength in the device became weaker for an LC cell with a larger thickness, which therefore called for larger voltages including higher V_th_ and V_sat_ values, to rearrange the mesogens. On the contrary, V_th_ and V_sat_ decreased with the increase in the ammonium salt concentration (B). Since the ammonium salt played the role of the electrolyte, the increased ammonium salt concentration could reduce the device resistance and enhance the ability of the AC electric field to induce mesogens in the device, thus lowering the voltage for optical switching. Contrast ratio (CR), as another important factor for the electro-optical characteristics of PDLC devices, was defined as the ratio of T_on_ over T_off_, which also reflected the size of LC microdroplets. The contrast ratio values for **1**, **2**, **5**, **6**, **7**, **8**, **9**, **10**, and **11** were graphed as shown in Figure 4d, which obviously exhibited the CR values of **5** (CR = 42.9) and **7** (CR = 36.7) were higher than the others.

**Figure 5:**
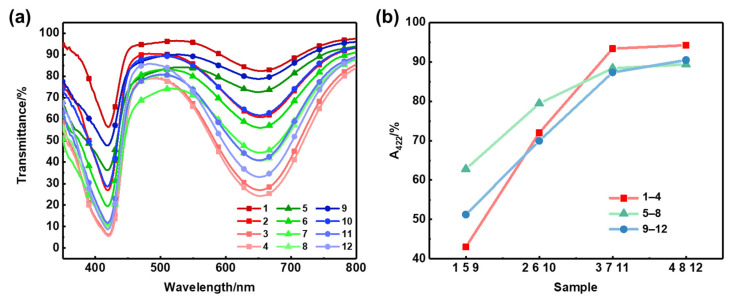
(**a**) Transmittance spectra and (**b**) the corresponding absorption at 422 nm (A_422_) for **1**–**12** with the application of a DC electric field.

The EC property of **1**–**12** was studied as well. The transmittance spectra of **1**–**12** were collected with a DC current of 4.5 V applied (Figure 5a). Considering that the opaque initial state could scatter light and affect the EC-induced absorption measurements, samples were kept in the isotropic state with transmittance normalized to 100%. Samples were divided into three groups according to the monomer concentration, namely, **1**–**4** in the first group with the monomer concentration of 33.4 wt%, **5**–**8** in the second group with the monomer concentration of 42.9 wt%, and **9**–**12** in the third group with the monomer concentration of 53.9 wt%. For samples in the same group, the absorption at 422 nm (A_422_) increased as the ammonium salt concentration raised and saturated when the ammonium salt concentration exceeded 25.0 wt% (Figure 5b). The maximized value of A_422_ could reach 94.2% for samples **3** and **4**. The effect of the ammonium salt on the absorption of EC PDLC was similar to that of 5CB. In addition, the absorption at 422 nm (A_422_) was not affected by the monomer concentration and the cell thickness.

**Figure 6:**
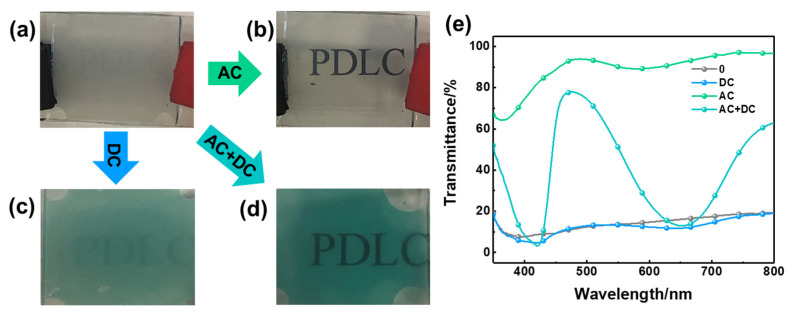
The physical appearance of the EC PDLC sample: (**a**) initial opaque colorless without no external electrical field, (**b**) transparent colorless with the application of an AC electric field (20 V), (**c**) opaque green with the application of a DC electric field (4.5 V), (**d**) transparent green with the application of both AC and DC electric fields, and (**e**) the corresponding transmittance spectra.

From the perspective of T_off_, T_on_, V_th_, V_sat_, CR, and A_422_, the electro-optical property of the EC PDLC device was comprehensively investigated by the orthogonal range difference method. When A (the weight ratio of the acrylate monomer mixture to 5CB) was in level II, the corresponding device presented better T_off_ and T_on_, the highest CR, and the smallest V_th_ and V_sat_. When B (the weight ratio of the ammonium salt to 5CB) belonged to level III, the corresponding device exhibited the lowest T_off_ and higher T_on_, lower V_th_ and V_sat_, and the highest CR, which also had saturated absorption and obvious coloration. When C (cell thickness) was in level II, the corresponding device demonstrated better T_off_ and T_on_, the smallest V_th_ and V_sat_, and higher CR. Consequently, we choose the weight ratio of the acrylate monomer mixture to 5CB of 42.9 wt%, the weight ratio of DDAB to 5CB of 25.0 wt%, and the cell thickness of 10 μm as the optimal constitution parameter for the fabrication of EC PDLC devices. In the end, a device with the optimal parameter was prepared and tested. It was able to display four different states by varying the external electrical field (Figure 6a–d). The device was initially in an opaque colorless state (Figure 6a). With an AC electric field of 20 V applied, the device turned into a transparent colorless state and reversibly returned to the initial state once the electric field was removed. With a DC electric field of 4.5 V applied, the device changed into an opaque green state and restore to its initial state after removing the electric field. Additionally, with AC (20 V) and DC (4.5 V) electric fields simultaneously applied, the device altered into a transparent green state and recovered after removing the electric field. The transmittance spectra associated with the status in Figure 6a–d were collected as shown in Figure 6e, which agreed with the corresponding visual appearance. Variations of mesogen species could produce similar results but different colors. For example, replacing 5CB with E7 resulted in a series of yellow-colored states instead of green-colored ones. To overcome the limitations of a single color, the specific work regarding multicolor EC PDLC devices with fabrication accessibilities to printing techniques, such as screen printing and ink-jet printing, is still under study.

## 4. Conclusions

In summary, a dual-responsive EC PDLC device was successfully fabricated by combing the PDLC technique with the reversible redox reaction between the benzene ring of mesogens and the cation of an ammonium salt under DC to form a colored complex. In the device, the mesogen 5CB not only played the role of scattering light in the form of microdroplets but also worked as a reactant for color development. Orthogonal experiments and range analysis methods were applied to evaluate the electro-optical characteristics and discoloration performance of those devices, which exhibited the fabrication parameter for the optimized device. The device presented four switchable states with the application of AC and DC electric fields. The AC electric field could change the light transmittance of the device while the DC electric field altered the device color. This work lays the foundation for realizing patterned multi-colored patterned displays and anti-counterfeiting via screen printing and inkjet printing techniques.

## Figures and Tables

**Table 1:** The factor-level table for orthogonal experiment design. A: the weight ratio of the acrylate monomer mixture to 5CB, B: the weight ratio of the ammonium salt to 5CB, and C: cell thickness (μm).

	A	B	C
I	0.334	0.112	5
II	0.429	0.177	10
III	0.539	0.250	15
IV	0.667	0.334	20

**Table 2:** Constitutions for PDLC devices generated by Software Minilab 21 and their corresponding PDLC feature.

Sample	A	B	C	PDLC ^a^
**1**	33.4 wt%	11.2 wt%	5	+
**2**	33.4 wt%	17.7 wt%	10	+
**3**	33.4 wt%	25.0 wt%	15	−
**4**	33.4 wt%	33.4 wt%	20	−
**5**	42.9 wt%	11.2 wt%	10	+
**6**	42.9 wt%	17.7 wt%	5	+
**7**	42.9 wt%	25.0 wt%	20	+
**8**	42.9 wt%	33.4 wt%	15	+
**9**	53.9 wt%	11.2 wt%	15	+
**10**	53.9 wt%	17.7 wt%	20	+
**11**	53.9 wt%	25.0 wt%	5	+
**12**	53.9 wt%	33.4 wt%	10	−
**13**	66.7 wt%	11.2 wt%	20	−
**14**	66.7 wt%	17.7 wt%	15	−
**15**	66.7 wt%	25.0 wt%	10	−
**16**	66.7 wt%	33.4 wt%	5	−

^a^: + and − referred to the presence and the absence of the PDLC feature, respectively.

## Data Availability

The data presented in this study are available on request from the corresponding author.
